# Reliability and validity of using telephone calls for post-discharge surveillance of surgical site infection following caesarean section at a tertiary hospital in Tanzania

**DOI:** 10.1186/s13756-017-0205-0

**Published:** 2017-05-08

**Authors:** Boniface Nguhuni, Pasquale De Nardo, Elisa Gentilotti, Zainab Chaula, Caroline Damian, Paola Mencarini, Emanuele Nicastri, Arnold Fulment, Alessandro Piscini, Francesco Vairo, Alexander M. Aiken, Giuseppe Ippolito

**Affiliations:** 1 Resource Centre for Infectious Diseases, Department of Internal Medicine, Dodoma Regional Referral Hospital, P.O Box 904, Dodoma, Tanzania; 2 ‘Lazzaro Spallanzani’ National Institute for Infectious Diseases-IRCCS, Rome, Italy; 3 Department of Infectious Diseases, Tor Vergata University, Rome, Italy; 4 Department of Obstetrics and Gynaecology, Dodoma Regional Referral Hospital, Dodoma, Tanzania; 5 College of Natural and Mathematical Sciences, University of Dodoma, Dodoma, Tanzania; 6 Department of Clinical Research, London School of Hygiene and Tropical Medicine, London, UK

**Keywords:** Caesarean section, Surgical site infection, Post-discharge surveillance, Phone call interview, Resource limited settings

## Abstract

**Background:**

Surgical site infection (SSI) is a common post-operative complication causing significant morbidity and mortality. Many SSI occur after discharge from hospital. Post-discharge SSI surveillance in low and middle income countries needs to be improved.

**Methodology:**

We conducted an observational cohort study in Dodoma, Tanzania to examine the sensitivity and specificity of telephone calls to detect SSI after discharge from hospital in comparison to a gold standard of clinician review. Women undergoing caesarean section were enrolled and followed up for 30 days. Women providing a telephone number were interviewed using a structured questionnaire at approximately days 5, 12 and 28 post-surgery. Women were then invited for out-patient review by a clinician blinded to the findings of telephone interview.

**Results:**

A total of 374 women were enrolled and an overall SSI rate of 12% (*n* = 45) was observed. Three hundred and sixteen (84%) women provided a telephone number, of which 202 had at least one telephone interview followed by a clinical review within 48 h, generating a total of 484 paired observations. From the clinical reviews, 25 SSI were diagnosed, of which telephone interview had correctly identified 18 infections; telephone calls did not incorrectly identify SSI in any patients. The overall sensitivity and specificity of telephone interviews as compared to clinician evaluation was 72 and 100%, respectively.

**Conclusion:**

The use of telephone interview as a diagnostic tool for post-discharge surveillance of SSI had moderate sensitivity and high specificity in Tanzania. Telephone-based detection may be a useful method for SSI surveillance in low-income settings with high penetration of mobile telephones.

## Introduction

Surgical site infection (SSI) is one of the most common healthcare-associated infections (HAI) [1, 2], especially in low and middle-income countries (LMIC) [3–5]. Caesarean section (CS) is among the most frequent surgical interventions in women worldwide [6, 7]. However, CS increases the risk of post-partum infections by 5 to 20-fold compared to vaginal delivery [8, 9]. Despite modern surgical techniques and antibiotic prophylaxis, SSI is still contributing significantly to morbidity, mortality and healthcare-associated costs [6, 10, 11]. Data from Tanzania report SSI incidences of 10.9, 24% and as high as 48% [6, 12, 13]. Many SSI occur after discharge from hospital, typically occurring between 5 and 10 days post-operatively. The burden of SSI is therefore liable to be underestimated by any HAI surveillance system that does not capture information after discharge from hospital [14, 15].

There is no scientific consensus on an ideal method of detecting SSI after discharge from hospital and different approaches have been employed in different settings [16–19]. However, there is emerging evidence that patients are able to diagnose and report their own SSI with an acceptable accuracy if prompted with the appropriate questions via telephone calls [14–16, 20]. Evaluations of such telephone-based methods in LMIC including comparison to a “gold-standard” reference diagnosis is very limited so substantial uncertainty remains about the reliability of these methods [21].

The aim of this study was to examine the feasibility, sensitivity and specificity of the use of a structured questionnaire administered through telephone interview as a method for detecting post-discharge SSI after CS. The survey was conducted at a tertiary hospital in Tanzania. We also sought to estimate the overall risk of SSI occurring in a 30-day post-operative period in this population.

## Methodology

### Study design

Prospective observational cohort study.

### Study site

Obstetrics and Gynaecology Department of the Dodoma Regional Referral Hospital (DRRH), Dodoma, Tanzania. The average number of women delivering at DRRH is around 1000 per month, with CS accounting for around 20% of deliveries.

### Period of study

Two month enrollment period from May to June 2015.

### Study population

All women admitted at DRRH labour ward who underwent CS were enrolled within 24 h of surgery and were followed up for 30 days for development of SSI, in accordance with the United States Centers for Disease Control and Prevention (CDC) criteria for diagnosis of SSI [20].

### Data collection and follow up

Data were retrieved from the patients using a structured questionnaire and through medical records. All women were asked to provide one or more mobile telephone numbers, either a personal phone and/or that of a relative for communication after discharge. Before discharge, women were educated on proper wound care at home and were asked to attend Makole Health Centre (MHC) for wound inspection, dressing and consultation with medical doctors at day 7 post-CS. Before this scheduled visit, all women who provided a mobile number were contacted on day 5 or 6 post-CS and a brief structured interview regarding the status of the wound was conducted. The telephone interview questions were related to signs and symptoms of infection including history of fever, pus discharge from the wound, pain or redness at the surgical wound, use of antibiotics or any drugs to support wound healing and presence of obvious gaping of the wound or protrusion of internal structures. Patients were also reminded to attend MHC within 48 h of the phone interview. The phone call was performed by a clinically-trained investigator or a registered nurse who had received appropriate training on the telephone interview questionnaire. The diagnosis of SSI was made using an algorithm based on the CDC classification system [22]. At the MHC follow-up clinic, patients were evaluated by different clinicians who were trained on the CDC criteria for diagnosis of SSI and unaware of the findings of the telephone interview. Phone call interviews were also performed on days 12 and 28 post-CS prior to additional scheduled visits on days 14 and 30 post-CS, respectively. Patients did not receive any kind of incentives or fare reimbursements to attend the clinic during the study.

### Data analysis

Data were analysed using STATA software (Version 12.0, STATA Corp, College Station, Texas, USA). To determine the sensitivity and specificity of phone calls, an analysis was performed using all paired observations of telephone call interview and clinician review (which was considered as the gold standard) that occurred within 48 h of each other. We performed the analysis using the paired observations at each separate time-point and at all the three time-points combined.

## Results

A total of 374 women who underwent CS during the study period were enrolled. Patients’ characteristics are shown in Table 1.

**Table 1:** Characteristics of all enrolled patients (*n* ^0^ = 374) and patients with both telephone interview and clinician review within 48 h (*n* ^1^ = 202)

Characteristics	*n* ^0^ = 374	(%)	*n* ^1^ = 202	(%)
Age in years				
Mean age: 26.3 (STD ± 6.5)				
< 20	89	23.8	28	13.9
20–34	227	60.7	141	69.8
≥ 35	51	13.6	29	14.3
Unknown	7	1.9	4	2.0
Residence				
Dodoma Urban District	274	73.3	158	78.2
Other districts	100	26.7	44	21.8
Education level				
No education	53	14.2	24	11.9
Primary education	152	40.6	92	45.5
Secondary education	102	27.3	50	24.7
Higher education	18	4.8	10	5.0
Unknown	49	13.1	26	12.9
Occupation				
Peasants	101	27.0	48	23.8
Petty business	75	20.0	50	24.7
Housewives	92	24.6	50	24.7
Formal employment	43	11.5	21	10.4
Others	34	9.1	17	8.4
Unknown	29	7.8	16	8.0
BMI				
Underweight (<18.5)	3	0.8	0	0.0
Normal weight (18.5–24.9)	137	36.7	61	30.2
Overweight (25–29.9)	110	29.4	76	37.6
Obesity (≥ 30)	60	16.0	40	19.8
Unknown	64	17.1	25	12.4
Type of Caesarean Section				
Elective	15	4.0	10	5.0
Emergency	359	96.0	192	95.0
Pre-incision antibiotics				
Yes	369	98.7	200	99.0
No	4	1.0	2	1.0
Not known	1	0.3	0	0.0

### Use of telephone calls in detection of SSIs

Three hundred and sixteen (84%) enrolled women provided a telephone number. Phone calls were successfully made on at least one occasion to 274 patients, representing 87% (274/316) of patients with a telephone number. Forty-two (13%) patients were not reachable after at least two attempts. Among the 274 successfully interviewed patients, 202 attended for a clinician review within 48 h of the interview at least once during the study. A total of 484 paired observations (telephone call interviews and clinician reviews) within 48 h of each other were generated across the three time-points. From all observation pairs, 18 telephone interviews identified an SSI, all sub-classified as “superficial”. In clinical review, 25 SSI were diagnosed corresponding to an SSI rate of 12.3%, including all 18 Superficial SSI identified by telephone interview, plus 7 further infections (4 Superficial, 2 Deep and 1 Organ/Space). When considering just single time-points, similar proportions of patient with SSI were identified at the day 7 and day 14 time-points, but no patients with SSI were identified at the day 30 time-point, either by telephone call or clinical review as shown in Table 2.

**Table 2:** The incidence of SSI by telephone call interview and clinician’s evaluation at different time-points

Telephone call time-point	D7	D14	D30	Combined
Total paired observations	187	156	141	484
SSI diagnosed by clinical review (gold standard)	14	11	0	25
SSI correctly detected by telephone interview	11	7	0	18
Incorrect detection of SSI by telephone interview (false positive test)	0	0	0	0
Sensitivity of phone call (95%CI)	79% (49.2–95.3)	64% (30.8–89.1)	N/A	72%(50.2–87.9)
Specificity of phone call (95%CI)	100% (97.9–100)	100% (97.5–100)	N/A	100%(99.2–100)
Positive Predictive Value (PPV)	100% (71.5–100)	100%(59–100)	N/A	100%(81.5–100)
Negative Predictive Value (NPV)	98.3%(95.1–99.6)	97.3%(93.3–99.3)	N/A	95.9%(96.9–99.4)

*Key: N/A- Not applicable*

Using this combined set of all paired observations, the sensitivity and specificity of telephone interviews against a clinical review gold-standard were 72% (95% CI 50.6–87.9) and 100% (95% CI 99.2–100), respectively while positive predictive value (PPV) and negative predictive value (NPV) were 100% (95% CI 81.5–100) and 95.9% (95% CI 96.9–99.4), respectively as presented in Table 2. Logistically, each telephone call interview required between 3 and 5 min and the direct cost was approximately US$0.50.

### Risk of SSI, based on different follow-up strategies

Out of 374 women, an overall SSI rate of 12.0% (*n* = 45) was observed and confirmed by clinical review. All SSI occurred after initial discharge from hospital which was typically within 10 days after CS. The median interval from surgery to clinical diagnosis was 8 days (IQR 7–11 days). Sixty eight percent (*n* = 254) of enrolled women attended the outpatient clinic at least once within 30 days post-CS, with complete loss to clinical follow-up occurring in 120 (32%) women. According to the CDC classification of SSI, 42/45 (93%) were Superficial, 2 (4.4%) were Deep and 1 (2.2%) was an Organ/Space infection. As shown in Table 3, we retrospectively determined what SSI risk might have been found by using partial elements of the telephone-based SSI surveillance. Based on these data, we find that combination of two phone calls at day 7 and day 14 would have detected almost 90% of SSI cases.

**Table 3:** Risk of SSI by using telephone based surveillance

SSI Surveillance	Total SSI (full cohort *n* = 374)	Implied risk of SSI in cohort %	Proportion of SSI detected
Inpatient detection only	0	0.0%	0%
Phone call at day7 only	23	6.1%	51%
Phone call at day14 only	17	4.5%	38%
Phone call at days7 and 14	40	10.7%	89%
Any SSI detected in study	45	12.0%	100%

## Discussion

The overall sensitivity and specificity of telephone call interview to detect SSI in comparison to direct clinical evaluation was 72 and 100%, based on 484 paired observations. The sensitivity was not statistically different at day 7 (79%) and day 14 (64%). No infections were detected by telephone call interviews or clinical reviews to detect SSI at day 30 post-surgery, suggesting that there is limited value for reviews, either clinical or telephone-based at this time-point.

Our estimates of the sensitivity and specificity of this method might have been affected by the 48 h interval between telephone interview and review by clinicians, since some infections may have become symptomatic at this time. This might lead to underestimation of the sensitivity. A substantial number of patients were not reachable by mobile phones, which means that the use of phone calls as a stand-alone tool would be unlikely to detect all SSI.

Some other studies have described a role for telephone call interviews for SSI surveillance in LMIC [13, 16, 21, 23]. Our study is, to our knowledge, the largest-ever direct evaluation of the role of telephone interview in SSI surveillance in an African country. The results from our study are similar to a smaller-scale research conducted in Kenya, which also observed moderate sensitivity and high specificity of phone calls as a stand-alone test for SSI [14]. However, telephone interview did not detect the “Deep” and “Organ/Space”” SSI that were detected in this study, which is potentially a cause for concern as these represent the most serious forms of SSI.

As in many other SSI studies [21, 24, 25], the post-discharge surveillance was affected by significant loss to follow up. The failure to attend the clinic might be related to the difficulty, distance and cost for women to reach the clinic. For clinical review-based methods of SSI surveillance, this means that an operating centre is liable to miss the occurrence of post-operative complication and hence underestimate local SSI risks. However, if SSI surveillance systems made greater use of telephone call-based methods, more accurate estimates might be obtained.

In Tanzania, as in many LMICs, there is now relatively high penetration of mobile phones for communication and other uses including electronic money transfers. This technology represents a significant opportunity for public health and disease surveillance systems. Amongst women of child-bearing age in Dodoma, 85% were able to provide a mobile phone number. However, there are also barriers to contacting by phone beyond ownership, which is reflected by our finding that only 87% of those providing a phone number were successfully contacted. As in other studies, the telephone call interviews were well received by both the patients and the healthcare workers].

The cumulative incidence of post-CS SSI in this study was 12% which is substantially reduced from an incidence of 48% described at DRRH in 2013 in a previous study [26]. Given the substantial loss to follow-up, this value may underestimate the true occurrence of post-operative risk of infection. This SSI rate is lower than typically reported in Tanzania, however is comparable to other studies conducted in LMICs but it is significantly higher than those typically reported in high-income countries (6,10,12,26)]. As observed in other similar studies, the commonest type of infection was Superficial [6, 10, 12, 27]. According to our data, the majority of SSIs were detected within ten days post-CS, indicating that contamination in the operating theatre is liable to be a significant underlying causal factor [6, 28].

## Conclusion

The occurrence of SSI represents a substantial risk after surgery in LMICs and appropriate detection of these events is difficult when patients have to travel a long distance to reach healthcare facilities. Case ascertainment of SSI can be achieved by a variety of methods and a combined approach can be considered. Potentially, the use of a structured questionnaire administered through telephone calls for post-operative surveillance might reduce loss to follow-up and improve the quality of surveillance data. However, although we found the specificity to be very high, further work is needed to explore how the sensitivity of telephone-based diagnosis and ability to make contact with patients by telephone could be further improved, especially in settings where few patients are able to attend for clinical review.

## Abbrreviations

CDC: Centres for diseases control and prevention
CS: Caesarean section
DRRH: Dodoma Regional Referral Hospital
HAI: Healthcare-associated infections
IQR: Inter quartile range
LMIC: Lower and middle-income country
MHC: Makole Health Centre
NPV: Negative predictive value
PPV: Positive predictive value
SSI: Surgical site infection
